# Validation of a High-Throughput Multiplex Genetic Detection System for *Helicobacter pylori* Identification, Quantification, Virulence, and Resistance Analysis

**DOI:** 10.3389/fmicb.2016.01401

**Published:** 2016-09-07

**Authors:** Yanmei Zhang, Fuju Zhao, Mimi Kong, Shiwen Wang, Li Nan, Binjie Hu, Michal A. Olszewski, Yingxin Miao, Danian Ji, Wenrong Jiang, Yi Fang, Jinghao Zhang, Fei Chen, Ping Xiang, Yong Wu, Hu Zhao

**Affiliations:** ^1^Department of Laboratory Medicine, Huadong Hospital affiliated to Fudan University Shanghai, China; ^2^Key Laboratory of Clinical Geriatric Medicine Shanghai, China; ^3^Research Center on Aging and Medicine, Fudan University Shanghai, China; ^4^Ningbo HEALTH Gene Technologies Co., Ltd Ningbo, China; ^5^Division of Pulmonary and Critical Care Medicine, Department of Internal Medicine, University of Michigan Health System and Research Service, VA Ann Arbor Healthcare System, Ann Arbor MI, USA; ^6^Digestive Endoscopic Center, Huadong Hospital affiliated to Fudan University Shanghai, China

**Keywords:** *Helicobacter pylori*, high-throughput multiplex genetic detection system, identification, quantification, virulence, resistance genes, mixed infection

## Abstract

*Helicobacter pylori* (*H. pylori*) infection is closely related to various gastroduodenal diseases. Virulence factors and bacterial load of *H. pylori* are associated with clinical outcomes, and drug-resistance severely impacts the clinical efficacy of eradication treatment. Existing detection methods are low-throughput, time-consuming and labor intensive. Therefore, a rapid and high-throughput method is needed for clinical diagnosis, treatment, and monitoring for *H. pylori*. High-throughput Multiplex Genetic Detection System (HMGS) assay was established to simultaneously detect and analyze a set of genes for *H. pylori* identification, quantification, virulence, and drug resistance by optimizing the singlet-PCR and multiple primers assay. Twenty-one pairs of chimeric primers consisted of conserved and specific gene sequences of *H. pylori* tagged with universal sequence at the 5′ end were designed. Singlet-PCR assay and multiple primers assay were developed to optimize the HMGS. The specificity of HMGS assay was evaluated using standard *H. pylori* strains and bacterial controls. Six clinical isolates with known genetic background of target genes were detected to assess the accuracy of HMGS assay. Artificial mixed pathogen DNA templates were used to evaluate the ability to distinguish mixed infections using HMGS assay. Furthermore, gastric biopsy specimens with corresponding isolated strains were used to assess the capability of HMGS assay in detecting biopsy specimens directly. HMGS assay was specific for *H. pylori* identification. HMGS assay for *H. pylori* target genes detection were completely consistent with the corresponding genetic background. Mixed infection with different drug-resistant isolates of *H. pylori* could be distinguished by HMGS assay. HMGS assay could efficiently diagnose *H. pylori* infection in gastric biopsy specimens directly. HMGS assay is a rapid and high throughput method for the simultaneous identification and quantification of *H. pylori*, analysis of virulence and drug resistance in both isolated strains and biopsy specimens. It could also be used to distinguish the mixed infection with different resistant genotype strains. Furthermore, HMGS could detect *H. pylori* infection in gastric biopsy specimens directly.

## Introduction

*Helicobacter pylori* (*H. pylori*), a micro-aerobic Gram-negative bacteria, is closely associated with a variety of gastroduodenal diseases, such as chronic superficial gastritis (CSG), chronic atrophic gastritis (CAG), peptic ulcer diseases (PUD), and gastric carcinoma (GC) (Venerito et al., 2016). More than half of the adult population worldwide was infected by this pathogen and the most serious incidence of infection was observed in East Asia (de Martel et al., 2013). The analysis of different virulence, gene-mutation related resistance and the bacterial load have been revealed to be very important for effective clinical diagnosis, treatment, and monitoring of *H. pylori* infection (Kusters et al., 2006; Proença-Modena et al., 2009; Yamaoka, 2010).

Currently, various detection methods are available in clinical practice, such as culture, histopathology, urea breath test (UBT), rapid urease test (RUT), serology, and PCR, but each has some disadvantages (Wang et al., 2015). To date, no single method incorporates simultaneous analysis of strain-specific features including identification, virulence characteristics, drug-resistance, quantification, and evaluation of multi-strain infections (Shukla et al., 2011). Thus, a rapid, accurate, and high-content quantitative assay is required to directly assist clinical diagnosis in tissue and improve the effectiveness of diagnosis and antibiotic treatment.

Previously, our team has developed a genetic analysis method using *16S rRNA* and *ureC* for *H. pylori* identification and quantification (Zhou et al., 2015). Here, we further explored and developed a new multiple genes detection assay. In this study, total 21 pairs of chimeric gene specific primers were designed to establish a high-throughput multiple genetic detection system (HMGS) for simultaneous bacterial diagnosis and identification of virulence and resistance genes. The specificity of HMGS assay was assessed using the standard *H. pylori* strains and bacterial controls. Six clinical isolates with known genetic background of target genes were detected to assess the accuracy of HMGS assay. Artificially mixed DNA from different wild type and mutant resistant isolates of *H. pylori* was used to evaluate the ability to distinguish mixed infections. Furthermore, gastric biopsy specimens with the corresponding isolated strains were used to evaluate the capacity of HMGS assay in detecting biopsy specimens directly.

## Materials and Methods

### Ethics Statement

The study has been approved by Ethics Committee for human studies of Huadong Hospital. The Ethics Approval Number: [2013]-077.

### Bacterial Strains and Clinical Specimens

The standard strains *H. pylori* ATCC43504, *Escherichia coli* (*E. coli*) ATCC8739, *Pseudomonas aeruginosa* (*P. aeruginosa*) ATCC35218, and *Acinetobacter baumannii* (*A. baumannii*) ATCC19606 were purchased from Shanghai Municipal Center for Disease Control & Prevention. Six clinical strains of *H. pylori* with known genetic background and *Campylobacter jejuni* (*C. jejuni*), *Klebsiellar pneumonia* (*K. pneumonia*), *Stenotrophomonas maltophilia* (*S. maltophilia*) were isolated and confirmed by their specific and conserved species genes. Two clinical antrum tissue specimens were obtained from patients who were diagnosed as *H. pylori* positive by C^14^ expiration test and one uninfected antrum tissue from a healthy control in the Huadong Hospital, Shanghai, China.

### Culture and DNA extraction

Each antrum biopsy specimen was placed in 0.9% sterile saline solution kept at 4°C and sent to the microbiology laboratory within 4 h. The biopsy specimens were homogenized using TissueLyser (Jingxin Co., Ltd., Shanghai, China) and plated on Columbia agar (OXOID Microbiology Products, Thermo Fisher Scientific Inc., Waltharm, MA, USA) supplemented with medium containing 8% sterile defibrinated sheep blood (Shunwei Biotech Co., Ltd., Shanghai, China) and 0.5% selective antibiotics (Sigma-Aldrich, USA) supplement. The cultures were kept under microaerophilic conditions (5%O_2_, 10%CO_2_, 85%N_2_) at 35°C for 3-7 days. The isolates were frozen in glycerin broth at -80°C and the remained homogenated specimens were kept in -80°C for DNA extraction. Total DNA from the standard strains, clinical isolates and *H. pylori* positive biopsy specimens was extracted with a bacterial genomic DNA extraction kit (Tiangen Biotech Co., Ltd., Beijing, China) following the manufacturer’s instruction. The extracts were eluted with 100 μL of DNase/RNase-free H_2_O (ddH_2_O). Concentration of each extract was determined using a ThermoNano drop 2000 spectrophotometer (Thermo Fisher Scientific Inc., Waltharm, MA, USA). The extracts were stored at -20°C for further analysis.

### Primer Design

The primer set for HMGS assay consisted of 21 pairs of chimeric primers, including identification gene *16S rRNA*, 10 main virulence-associated genotypes *cagA, vacA s1, vacA s2, vacA m1, vacA m2, iceA1, iceA2, luxS, dupA*, and *oipA*, four drug resistance genes *23S rRNA* (A2143G), *rdxA* (C148T), *gyrA*(C261A/G), and *pbp1A* (A1777G), quantification gene of *ureC*, and human tissue internal control *beta-globin* gene which induced to normalize the specific gene expression. In addition, it also has one pair of universal primers. All chimeric primers consisted of a gene-specific sequence tagged with a universal sequence at the 5′ end to ensure the specific and equivalent amplification. The forward universal primers were Cy5-labeled at the 5′ end of the sequence. Hundreds of sequences for each target from different region of the world, especially for those from Asia were downloaded from the National Center of Biotechnology Information (NCBI) and analyzed using Vector NTI to identify the homolog regions. The primers were designed in highly conserved regions using DNA star software (DNASTAR Inc., Madison, WI, USA) and Primer Premier 5.0 software (Premier Biosoft International, Palo Alto, CA, USA). The specificity of each single pair of primers was verified by conventional PCR and sequencing. These gene-specific primers were designed and optimized following several main principles: homogeneity, the length of amplification product is 150-350 bp, at least three bases difference between each amplicon, no significant dimers between different primers, and no non-specific products by each pair of gene-specific primers (Robertson and Walsh-Weller, 1998; Bruce, 2003). The primer sequences (Sunny Biotech Co., Ltd), the size of the resulting amplicons, and their target genes were listed in **Table 1**.

**Table 1:** Primer sequences and product size in HMGS.

Primer	SNP	Sequence (5′-3′)	Accession number	Size (bp)
Gene of identification	*16S rRNA*	F: AGGTGACACTATAGAATAATTACTGAAGCTGATTGCGC	KC525436.1	147
		R:GTACGACTCACTATAGGGACTGGAGAGACTAAGCCCTCC
	*ureC*	F: AGGTGACACT AT AGAAT AAGCCACAACCCTTTTGAAGA	M60398.1	138
		R:GTACGACTCACTATAGGGATCATGAAAGATTTCTTCAATCGCT

Gene of virulence	*cagA*	F: AGGTGACACTATAGAATAGATC**K**TTTTGATGGGACACC	AHO 13328.2/ AHO 13327.2	210
		R: GTACGACTCACTATAGGGACAAAAATCCTACCAAAAAGAATCAGT
	*vacA s1*	F: AGGTGACACTATAGAATACTGCTTGAATGCGCCAAAC	KC665712.1/HQ287752.1	299
		R: GTACGACTCACTATAGGGAATGGAA**W**TACAACAAACACACC
	*vacA s2*	F: AGGTGACACTATAGAATACTGCTTGAATGCGCCAAAC	AY049006.1 /AB190976.1	327
		R: GTACGACTCACTATAGGGAATGGAA**W**TACAACAAACACACC
	*vacA m1*	F: AGGTGACACTATAGAATAGTTTAGAAACTGGCAC**Y**AGGTCAA	LC068288.1/LC068427.1/LC068302.1	282
		R: GTACGACTCACTATAGGGAAAATTGGCTATAATCCATGAC**Y**G
	*vacA m1*	F: AGGTGACACTATAGAATATTGCTTGATGGCCTGCATT	LC068424.1	166
		R: GTACGACTCACTATAGGGAGCAAGCATGGATTATGGTAAGGA
	*iceAl*	F: AGGTGACACTATAGAATACCAGGAATTTTTSTTGCATCAA	AF239991.1/AP012600.1/CP010436.1	234
		R: GTACGACTCACTATAGGGAGGCAA**Y**TCTGAAAACACTCA
	*iceA2*	F: AGGTGACACTATAGAATAACTTTACCCTTTGATGTGGTTAC	JQ808070.1/AF 176822.1	171/277
		R: GTACGACTCACTATAGGGATGTA**R**TTAAAGTCGTTAATGGCAA
	*dupA*	F: AGGTGACACTATAGAATAGTGGGGTA**D**ATAATCACTTGAGA	KM245039.1 /AB739570.1/AB739567.1	187
		R: GTACGACTCACTATAGGGAACCTATATCGCTAACGCACT
	*oipA*	F: AGGTGACACTATAGAATACCAATCACAAGCCCTGAAGAT	KJ816694.1	317
		R: GTACGACTCACTATAGGGAATTATAGGGTTTAGGCACTCTCTT
	*luxS*	F: AGGTGACACTATAGAATAACACCAAAGTCAAAGCCCCT	DQ777750.1	242
		R: GTACGACTCACTATAGGGACCCATAGGCGACCAATCCA**Y**
Gene of resistance	*23S rRNA*	F: AGGTGACACT AT AGAAT AGGTGGTATCTCAAGGATGGC	KT958602.1 /KT958601.1	175
	A	R: GTACGACTCACTATAGGGAAACCGCGGCAAGACGGGA		180
	G	R: GTACGACTCACTATAGGGAGATCTAACCGCGGCAAGACGGCG	
	*rdxA*	F: AGGTGACACT AT AGAAT ACACTCTAAC**Y**TT AT AAGACTCYGG**R**TA	AP014712.1/AP014711.1 /CP007603.1	253
	C	R: GTACGACTCACTATAGGGACGCCAAGCTCTTACAACACCC		2598
	T	R: GTACGACTCACTATAGGGAACTATCGCCAAGCTCTTACAACACTT
	*pbp1A*	F: AGGTGACACT AT AGAAT ATTTGGGGACATCAAACTTTCTT	CP011482.1 /AB075016.1 /CP011485.1/AB128022.1	154
	A	R: GTACGACTCACTATAGGGACGAC**Y**ATTRGCAAAGGAGCAA		159
	G	R: GTACGACTCACTATAGGGATAACACGAC**Y**ATT**R**GCAAAGGAGCTC
	*gyrA*	F: AGGTGACACT AT AGAAT AAAGGTTAGGCAGACGGCT	KT198991.1/JX944047.1/KC864784.1 /CP011483.1	306
	C/T	R: GTACGACTCACTATAGGGACACCCCCATGGCGATAT**Y**		311
	G/A	R: GTACGACTCACTATAGGGATTAACCACCCCCATGGCGATAG**R**

Housekeeping gene	*β-globin*	F: AGGTGACACT AT AGAAT ACTCTT ATCTTCCTCCCACAGCT	KR028331.1	199
		RGTACGACTCACTATAGGGAAGAAAGCGAGCTTAGTGATACT

*The highlighted base corresponds to degenerate bases as follows: K(G/T), W(A/T), Y(C/T), S(G/C), R(A/G), D(G/T/C).*

### Singlet-PCR Assay

The singlet-PCR assay was conducted using DNA templates verified by sequencing to evaluate the specificity of each pair of pathogen-specific primers and to ascertain the actual fragment size of each amplicon. The singlet-PCR assay was developed as follows: The singlet-PCR assay contained 0.5 μL of DNA, 12.5 μL of 2× buffer (Ex Taq polymerase, Mg^2+^, dNTP), 20 μM each of the forward universal primer and reverse universal primer, and 1 μM each of the forward chimeric primer and reverse chimeric primer. ddH_2_O was added to the PCR reaction to a final volume of 25 μL. The PCR mixture was incubated at 95°C for 5 min, followed by two steps with different annealing temperatures: step one, 12 PCR cycles of 30 s at 95°C, 30 s at 60°C, and 25 s at 72°C; step two, 20 cycles at 95°C for 30 s, 50°C for 30 s, and 72°C for 25 s; 5 min at 72°C, before then being held at 4°C in a thermal cycler. After amplification, 1 μL of PCR product that was diluted 10-fold was added to a 38.75 μL of specimens loading solution, along with 0.25 μL of DNA size standard-400 (GenomeLab GeXP Start Kit; Beckman Coulter, USA). Finally, a drop of mineral oil was added after blending. The GeXP Genetic Analysis System (Beckman Coulter, USA) was then used to analyze PCR products based on size separation using high-resolution capillary gel electrophoresis. The peak height for each PCR products was reported in the electropherogram and the reaction was considered positive when the dye signal was greater than 2,000 relative fluorescence units (rfu). ddH_2_O was used as a negative control throughout the assay.

### Establishment and Optimization of HMGS

The multiple primers and reaction factors were further optimized in one HMGS assay reaction. The multiplex quantitative PCR was performed in a mixture containing MgCl_2_ solution, PCR buffer, forward universal primers, reverse universal primers, Taq polymerase, DNA template, and ddH_2_O. In details, the reaction system was optimized as follows: All primers were added to the reaction system with the same concentration and the volume. After amplification, PCR products were prepared for fragment analysis using GeXP Genetic Analysis System following manufacture suggested protocols. One microliter of PCR product was added to 30 μL of specimens loading solution along with 0.5 μL of DNA Size Standard 400 (AB Sciex, Inc., USA). The mixture was added to a 96-well plate and was loaded onto a GeXP Genetic Analysis System for capillary electrophoresis and fragment separation. The peaks were initially analyzed based on the sizes of the appropriate amplified products. The peak height for each gene was reported in the electropherogram and the dye signal intensity was measured by fluorescence spectrophotometry in rfu (relative fluorescence unit). Then, the concentrations of primers were adjusted to optimize signal intensity for each target to be detected from 2,000 to 175,000 rfu. If a signal peak was too high, the corresponding concentration would be reduced until the peak intensity was moderate. If a signal peak was too low or not detectable, the primer concentration would be increased to enhance the signal intensity. Additionally, the annealing temperature was optimized through temperature gradient descent method (chimeric primers from 50 to 65°C). Meanwhile, other factors of reaction conditions were also optimized. The main optimized principle was to keep all amplicons with similar amplification efficiency and presenting the gene-specific target amplicon without cross-amplification. Here, the ratio and concentration of primers were critical. Meanwhile, other parameters of the reaction conditions such as Tm value, reaction time and temperature were also important and were systematically optimized. HMGS protocols referred to the instruction of GeXP and previously reported paper (Zhou et al., 2015).

### Artificial Mixture

To mimic clinical phenomenon in which patients infected different drug-resistant types of *H. pylori* simultaneously, four pairs of isolated strains from biopsy specimens with known genetic background, with mutation sites in *23S rRNA* (A2143G), *rdxA* (C148T), *gyrA* (C261A/G), and *pbp1A* (A1777G) were chosen, and DNA from the wild type and mutant resistant strains were artificially mixed together in equal concentrations in HMGS assay. In addition, DNA extracts from clarithromycin resistant isolated strain with mutation site in *23S rRNA* (*23S rRNA*_G) and from the wild type strain (*23S rRNA*_A) were chosen to mix together using following template ratio: 1:8, 1:4, 1:2,1:1, 2:1, 4:1, and 8:1. HMGS assay was performed using the mixed DNA.

### Specificity, Sensitivity, and Accuracy of HMGS Assay

To determine if optimized HMGS maintained high specificity for *H. pylori* detection, HMGS assay readout of a standard *H. pylori* stain (ATCC43504), ddH_2_O, and six control bacterial species were tested in HMGS platform. Our control bacterial species included: *E. coli, P. aeruginosa, A. baumannii, S. maltophilia, K. pneumoniae*, as well as *C. jejuni* which has very similar growth characteristics with *H. pylori*. To further confirm the specificity of HMGS in complicated environment, we have mixed six control bacterial species *C. jejuni, E. coli, P. aeruginosa, A. baumannii, S. maltophilia, and K. pneumoniae* DNA (0.8 ng/μL of each bacterial specie) with and without *H. pylori* DNA (0.4 ng/μL of each bacterial specie) in PBS and then detected by HMGS assay. The sensitivity of 21 primer pairs HMGS was conducted by eight serial double dilutions of *H. pylori* DNA in PBS from the concentration of 0.4 ng/μL to 3.125 × 10^-3^ ng/μL The HMGS limit of detection was set by the criteria that the all *H. pylori* specific signals would read above 2,000 rfu. Total six clinical isolates with known genetic background of target genes verified by Sanger sequencing and three antrum biopsy specimens (two cases from the patients with *H. pylori* infection and one from uninfected healthy control) were used to verify the accuracy of HMGS.

### Statistical Analysis

Statistical analysis of the linear correlation between the peak areas and the template ratios of wild type and mutant drug resistant isolates was carried out using SPSS version 17.0 software.

## Results

### Individual Primers Selected for HMGS Were Verified by Singlet-PCR Assay

**FIGURE 1:**
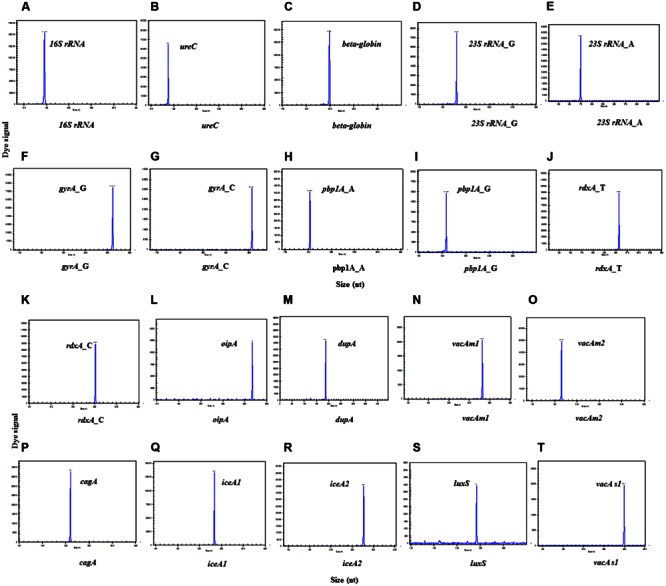
**Singlet-PCR assay.** The *X*-axis indicates the actual PCR product size, and the *Y*-axis indicates the dye signal. **(A-T)** Showed the results of the amplification of genes, *16S rRNA, ureC, beta-globin, 23S rRNA*_G, *23S rRNA*_A, *gyrA*_G, *gyrA*_C, *pbp1A gyrA*_A, *pbp1A gyrA*_G, *rdxA*_T,, *rdxA*_C,*oipA, dupA, vacA m1, vacA m2, cagA, iceA1*,*iceA2, luxS*, and *vacA s1*, respectively. Note that all gene targets were specifically amplified without nonspecific amplification by singlet-PCR assay.

The amplicon sizes for the target genes were as follows (**Figures 1A–T**): identification gene*16S rRNA*: 147 bp; quantification gene *ureC*: 138 bp; internal control gene *beta-globin*: 199 bp; 10 important virulence genes *cagA*: 210 bp, *dupA*: 187 bp, *luxS*: 242 bp, *vacA s1*: 299 bp, *oipA*: 317 bp, *vacA m1*: 282 bp, *vacA m2*: 166bp, *iceA1*: 234 bp, and *iceA2*: 171bp/277 bp; four main resistance genes *23S rRNA*(A2143G): 175 bp/180 bp, *rdxA*(C148T): 253 bp/258 bp, *pbp1A*(A1777G): 154 bp/159 bp, and *gyrA*(C261A/G):306 bp/311 bp, respectively. The result showed that all target genes were specifically amplified without unintended products by their corresponding primers.

### The Optimization of HMGS Conditions Allowed for Efficient Use of Multiplex Assay with High-Resolution Using Isolates

**FIGURE 2:**
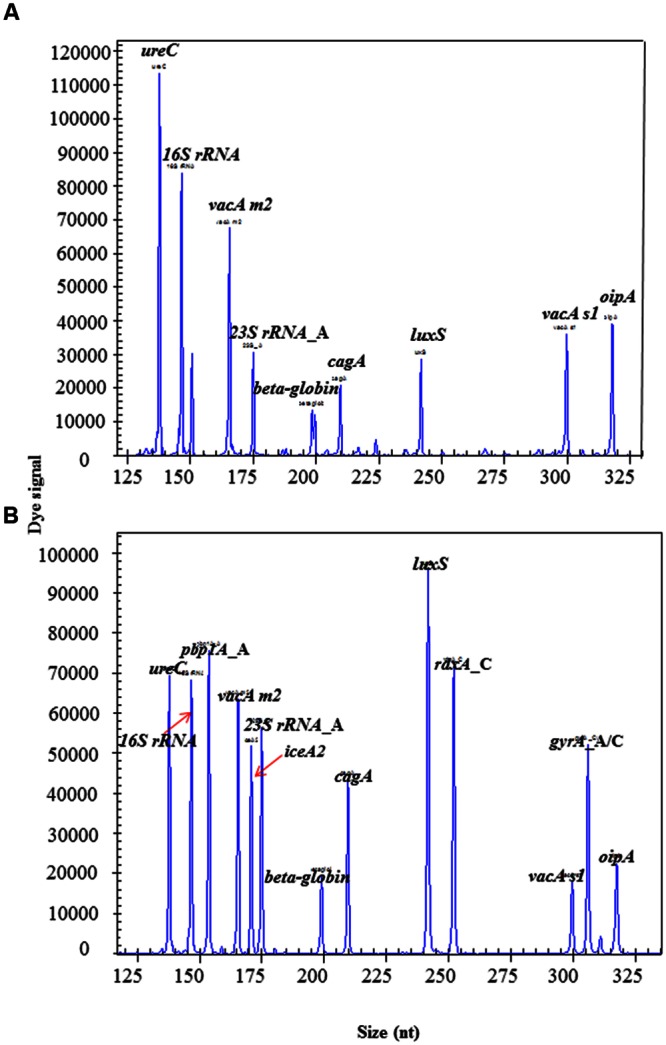
**The optimization of HMGS assay for *H. pylori* detection. (A)** The HMGS assay result showed specific gene from left to right was as follow: *ureC, 16S rRNA, vacA m2, 23S rRNA*_A, *beta-globin, cagA, luxS, vacA s1*, and *oipA*. **(B)** The targets genes from left to right are as follow: *ureC, 16S rRNA, pbp1A, vacA m2, 23S rRNA*_A, *iceA2, beta*-*globin, cagA, luxS, rdxA*_C, *vacA s1, gyrA*_C, and *oipA*. Note that the specific genes *pbp1A, iceA2*, and *gyrA*_C showed up and all the intensity of amplification signals for target genes moderated to the middle range after optimization.

The intensity of amplification signals for different targets varied up to 100-fold when all primers were used at the same concentration (**Figure 2A**). Therefore, the primer concentrations for genes with saturated peaks, such as *16S rRNA* and *ureC*, were reduced to reach a moderate signal levels. On the other hand, the primer concentrations for genes with very low peaks, such as *dupA, gyrA*, and *iceA1*, were increased two- to threefold to significantly enhance the signal levels. The overall signal intensity for all targets was moderate to the middle range in HMGS assay after optimization (**Figure 2B**).

### HMGS Assay is Specific for *H. pylori* Identification

**FIGURE 3:**
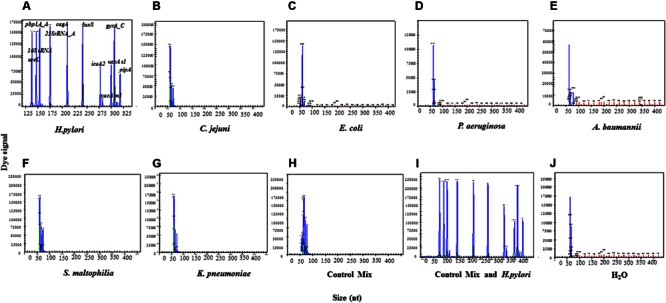
**The specificity of the HMGS assay. (A)** The standard *H. pylori* strain (ATCC43504) was used as positive control. **(B)** *C. jejuni*, **(C)** *E. coli*, **(D)** *P. aeruginosa*, **(E)** *A. baumannii*, **(F)** *S. maltophilia*, **(G)** *K. pneumoniae*, **(H)** control mix, **(I)** control mix and *H. pylori*, and **(J)** ddH_2_O were used as controls. *16S rRNA* had a specific peak at 146 bp and *ureC* had a specific peak at 137 bp for standard *H. pylori* strain. No specific peak was found in all negative controls. The peaks in **(B)** to **(H)** and **(J)** around 60 bp were primer dimers, excluded according to the standards provided by the manufacturer.

The *H. pylori* standard strain ATCC43504 showed specific amplification signals of *H. pylori* identification genes *16S rRNA* and *ureC* (**Figure 3A**), while other six control bacterial species including *C. jejuni, E. coli, P. aeruginosa, A. baumannii, S. maltophilia, K. pneumoniae*, and ddH_2_O did not produce any signals that would indicate non-specific amplification (**Figures 3B–F**). Furthermore, the control bacteria mix did not display any *H. pylori* specific signals, while the control mix with *H. pylori* showed all the specific signals (**Figures 3H,I**). The above results demonstrated that the newly established and optimized HMGS assay was highly specific for *H. pylori* identification. The limit of sensitivity for HMGS simultaneous detection of all genes using 21 primer pairs was 6.25 × 10^-3^ ng/μL while over 80% of genes were still above the detection limit at 3.125 × 10^-3^ ng/μL dilution of *H. pylori* DNA diluted in PBS.

### HMGS Can Simultaneously Detect Virulence and Drug Resistance Genes of *H. pylori*

**FIGURE 4:**
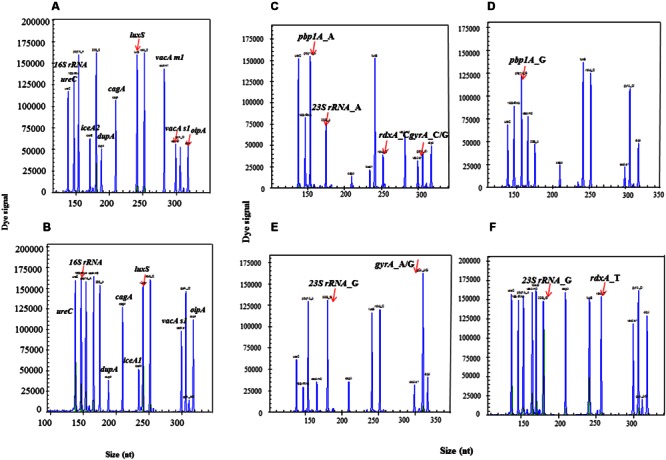
**The detection of identification, virulence and resistance genes by HMGS assay.** All the target genes could be efficiently detected by HMGS, including identification genes *16S rRNA* and *ureC* **(A-F)**, virulence genes *cagA, dupA, luxS, vacA s1, oipA, vacA m1*, and *iceA2* (A), *cagA, dupA, luxS, vacA s1, oipA*, and *iceA1* **(B)**, resistance genes *pbp1A* (A1777G) **(C,D)**, *23S rRNA* (A2143G) **(C,E)**, *gyrA*(C261A/G) **(C,E)**, and *rdxA* (C148T) **(C,F)**. Note that different isolates showed at the individual panels expressed distinct sets of identification, virulence and resistance genes, clearly identified by HMGS.

Six clinical isolates with known genetic background of target genes were used to evaluate the accuracy of HMGS assay. All the specific amplification signals were observed including identification gene *16S rRNA*, quantification gene *ureC*, internal control *beta-globin*, 10 main virulence-associated genes *cagA, vacA s1, vacA s2, vacA m1, vacA m2, iceA1, iceA2, luxS, dupA*, and *oipA*, and four drug resistance genes *pbp1A, gyrA, 23SrRNA, rdxA* (**Figure 4**). These results demonstrated that the optimized HMGS assay for target genes of *H. pylori* were completely consistent with the corresponding genetic background.

### HMGS Assay Allowed for Detecting Mixed Infection with Drug Resistant Mutant and Wild Type Isolates in a Wide Range of Proportions

**FIGURE 5:**
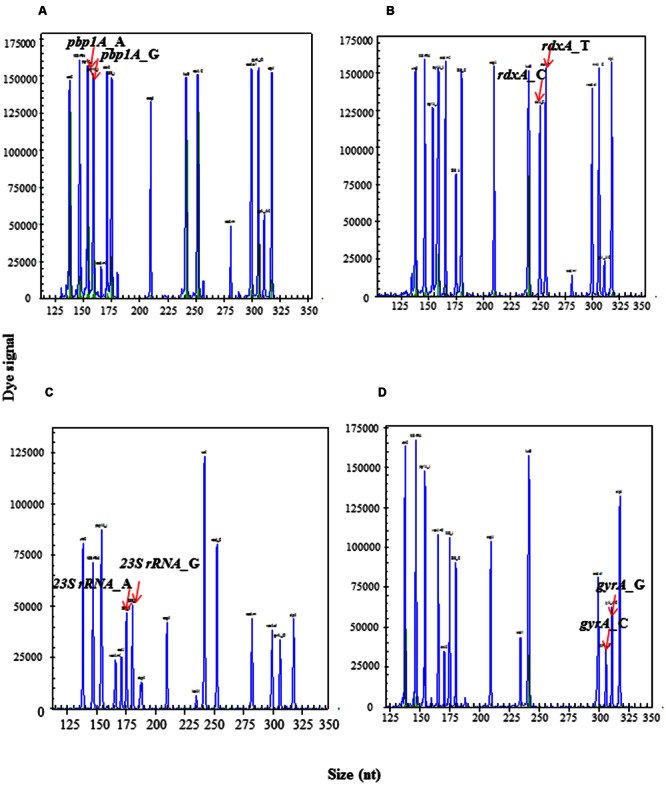
**Artificial mixture of wild type and resistant mutant isolates detected by HMGS assay.** The multiplex assay was carried out with artificial mixed wild type and resistant mutant templates for **(A)** *pbp1A*(A1777G), **(B)** *rdxA*(C148T), **(C)** *23SrRNA* (A2143G), and **(D)** *gyrA*(C261A/G), respectively. Note that mixed infection of wild type and mutant isolates could be accurately distinguished at a relatively broad range of proportions by HMGS assay.

**FIGURE 6:**
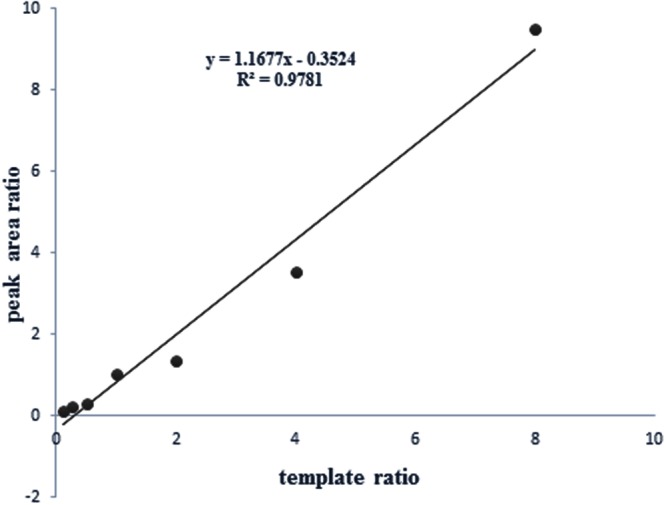
**Correlation between the peak areas and the template ratios of the wild type and mutant drug resistant isolates.** A near-identity (*R*^2^= 0.987) linear correlation between the peak areas and the template ratios showed that wild type and mutant isolates could be accurately quantify by HMGS assay at relatively broad range of proportions of the mixed infection.

The specific and distinct amplification signals for mixture templates with wild type and mutant of *H. pylori* were shown in **Figure 5**. These data demonstrated that HMGS assay could successfully detect and differentiate mixed infection with wild type and mutant resistant genes of *H. pylori* isolates. We perform additional test to further explore the possibility to detect the relative contribution of wild type strain and resistant mutant in mixed infections. DNA templates from CLA-resistant mutant clinical isolate (*23S rRNA*_G) and wild type isolate (*23S rRNA*_A) were prepared and amplified using following ratio: 1:8, 1:4, 1:2,1:1, 2:1, 4:1, and 8:1 per reaction in the HMGS assay. The relative magnitude of signals from the wild type and mutant clinical strains corresponded to the ratio of the mixed template. A near-identity (*R*^2^= 0.987) linear correlation between the peak areas and the template ratios has been found. These results showed that wild type and mutant isolates could be accurately quantify by HMGS assay at relatively broad range of proportions of the mixed infection strains (**Figure 6**).

### Optimization of HMGS Conditions Allowed for Efficiently Detecting of *H. pylori* Directly in Gastric Biopsy Specimens

**FIGURE 7:**
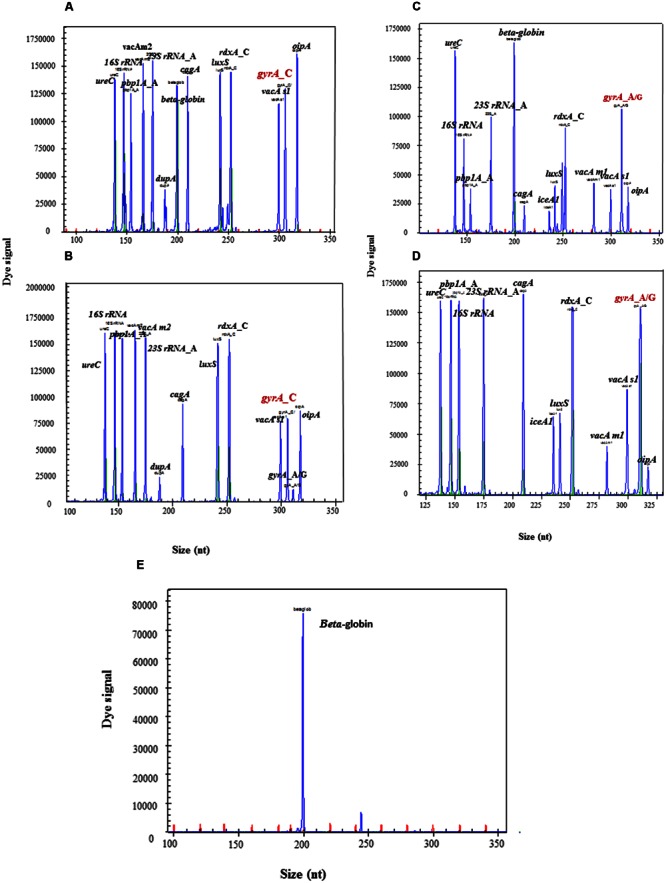
**Detection of antrum biopsy specimens and corresponding strains by HMGS assay.** The expected amplification peaks appeared in the wild type *gyrA_*C biopsy specimen **(A)** and its corresponding LEV-susceptible isolated strain **(B)**. The expected amplification peaks appeared in the mutant *gyrA_*A/G biopsy specimen **(C)** and its corresponding LEV-resistant isolated strain **(D)**. Only *beta*-*globin* peak appeared in uninfected biopsy specimen **(E)**.

Wild type and mutant resistant gene readouts of HMGS assay in biopsy specimens were completely consistent with their corresponding isolated strains. The peaks of *gyrA_*C were simultaneously showed in the wild type gastric biopsy specimen as well as in its corresponding LEV-susceptible isolates (**Figures 7A,B**), and the peaks of *gyrA_*A/G were showed in the mutant gastric biopsy specimens and its corresponding LEV-resistant isolates (**Figures 7C,D**). Meanwhile, all *H. pylori* specific peaks and human internal control gene beta-globin were detected in two infected biopsy specimens (**Figures 7A,C**), while only *beta-globin* was detected in uninfected biopsy specimens (**Figure 7E**). Thus, these results showed an excellent concordance between gastric biopsy specimens and corresponding isolated strains, documenting that HMGS assay could efficiently diagnose *H. pylori* infection and analyze the virulence and drug resistance in gastric biopsy specimens directly.

## Discussion

*Helicobacter pylori* is an important pathogen causing major gastroduodenal disease for which rapid non-culture based identification assays are needed (Graham, 1998). Virulence factors contribute to the development of severe complications in *H. pylori* infections and become predictors for risk of gastric atrophy, intestinal metaplasia and other severe clinical outcomes (Shiota et al., 2013). The severity of clinical manifestations is associated with bacterial load (Kusters et al., 2006). Meanwhile, the growing *H. pylori* drug resistance in Asia countries severely influences the efficacy of eradication treatment, which required drug resistance analysis before treatment (Katelaris, 2009; Cammarota et al., 2012; Alfizah et al., 2014; Smith et al., 2014). Therefore, it is important to analyze the virulence, bacterial load and resistance profiles during the identification of *H. pylori*. The traditional methods such as histology, culture, RUT, UBT as well as serology are available for the detection of *H. pylori*, and each method has its usefulness and limitations in different clinical situations (Hirschl and Makristathis, 2007; Khalifehgholi et al., 2013; Wang et al., 2015). The published guidance proposed molecular methods, like PCR sequencing, real-time (RT-PCR) PCR, and dual-priming oligonucleotide (DPO-PCR) should be introduced as alternatives for conventional *H. pylori* detection (Lehours et al., 2011; Malfertheiner et al., 2012) due to their high sensitivity and accuracy compared with conventional methods. However, these molecular methods are relatively expensive and could not detect multiple genes at a time (Lee et al., 2014; Wang et al., 2015). At present, there were no existing molecular methods that could simultaneously identify and quantify *H. pylori*, analyze the virulence and drug-resistance genes in single-infection and mixed infection condition. Thus, it is urgent to explore a rapid, effective, and high-throughput molecular technique to diagnose, treat, and monitor *H. pylori* infection. Previously, our team has established a genetic method using *16S rRNA* and *ureC* for *H. pylori* identification and quantification (Zhou et al., 2015). The sensitivity, specificity, positive predictive value (PPV) and negative predictive value (NPV) were comparable to other conventional methods, such as culture, RUT and histopathology. In this study, we incorporated this readout into a newly developed and optimized HMGS assay, introducing 21 newly designed primer pairs to simultaneously detect the identification gene, 10 virulence genes, 4 drug resistance genes, quantification genes *ureC* of *H. pylori* and internal control gene *beta-globin* of human tissue to normalize the specific gene expression.

Currently, some identification methods for *H. pylori* such as culture, histology, and RUT are available in clinical diagnosis but each of these methods has some limitations (Wang et al., 2015). The culture of gastric mucosa biopsy specimens is generally considered as the gold standard for the identification of *H. pylori*. However, culture is time consuming (Hachem et al., 1995; Cuchi et al., 2002), and its sensitivity and specificity are restricted with the technical difficulties, incubation environment and low bacterial loads (Cover, 2012). Histopathology has high sensitivity and specificity but the choice of staining methods and the bacterial load could directly impact the histological assessment (Christensen et al., 1992; Atherton et al., 1996; El-Zimaity et al., 1996). Besides, RUT is also susceptible to bacteria loads (Leodolter et al., 2001). In present study, we simultaneously detected the identification gene, virulence and resistance genes in six clinical isolated strains with known genetic background (**Figure 3**) and the results showed an excellent concordance with sequencing. In addition, specificity of the HMGS assay was examined using the standard *H. pylori* strain (ATCC43504), and six bacterial controls. The result showed that only standard *H. pylori* strains had specific amplification signals in *16S rRNA* and *ureC* genes at the size of 146 and 137 bp. However, neither *H. pylori* identification genes nor any of the other *H. pylori*-specific amplification signals appeared in HMGS conducted on control bacteria and negative control of ddH_2_O specimens. This result demonstrated that the optimized HMGS assay was highly specific to detect *H. pylori* (**Figure 6**). Therefore, this novel HMGS assay is a highly rapid, specific and effective technique compared with culture, histopathology and RUT.

Virulence factors have been reported to contribute to the development of *H. pylori* related-diseases and assist to diagnose in *H. pylori* infection (Proença-Modena et al., 2009). The specific functions of the virulence-associated factors of *H. pylori* is complicated, and its outcome likely affected by different combinations of variable regions of the virulence genes and influence of “local factors” in each studies (Shiota et al., 2013; Yamaoka and Graham, 2014; Sgouras et al., 2015). For example, there are variations in the functional vacA gene signal regions (s1 and s2) and middle regions (m1 and m2 in different *H. pylori* strains. Individuals infected with s1 or m1 *H. pylori* strains have an increased risk compared with individuals infected with s2 or m2 strain. Additionally, “i” region plays a role in the development of peptic ulcer in people in Iraq and Italy (Yamaoka, 2010); however, in a study of the patients from East and Southeast Asia, association between the region and disease has not been detected (Shiota et al., 2013). The immunoassay could be used to detect the virulence factors of *H. pylori*. However, immunoassay is relatively low-throughput in a single reaction. Especially, there is a window phase between the timing of *H. pylori* infection and antibody producing, which may lead to the high false negative rate (FNR) in clinical diagnosis of *H. pylori* infection. By using of HMGS assay, we here detected and analyzed 10 main virulence factors simultaneously (**Figure 4**). While understanding interactions between hosts and microbes expressing various combinations of virulence gene is very complex and will require an amount of data and system biology approach to analyze, we anticipate that our rapid, accurate, and high-throughput multiple virulence gene analysis will be a useful tool in gathering such information for further analysis. We believe that such information will enable us to identify the high-risk groups among carriers of *H. pylori*.

The most common antibiotics for *H. pylori* treatment are clarithromycin (CLA), metronidazole (MTZ), amoxicillin (AMX), and levofloxacin (LEV) according to the guidelines (Wolle and Malfertheiner, 2007; Gao et al., 2010; Yu et al., 2011; Malfertheiner et al., 2012). However, the growing drug resistance has seriously impacted the efficacy of eradication treatment, which emphasized the need of resistance analysis before antibiotic treatment. Conventional detection methods for *H. pylori* drug resistance are culture-dependent, time-consuming, low-throughput and could not distinguish mixed infection. *H. pylori* drug resistance was related to specific gene mutations (Gerrits et al., 2006; Tanih and Ndip, 2013). In our study, the four wild-type genes and their main gene mutation sites of *23S rRNA* (A2143G), *rdxA* (C148T), *gyrA* (C261A/G), and *pbp1A* (A1777G) were selected for HMGS to assess CLA, MTZ, LEV, and AMX resistance. We demonstrated that the HMGS assay performed on isolated strains was fast, effective and accurate for simultaneous analysis for drug resistance genes (**Figure 4**). Notably, the possibility of mixed infection with different drug resistant *H. pylori* strains increases the difficulty of differential diagnosis and individual eradication therapy. Here, we explored HMGS performance in detecting mixed infection using the artificial mixture with the wild type and mutant resistant genes, which showed the specific and distinct amplification signals for wild type and mutant type of *H. pylori* (**Figure 5**). Additionally, a linear correlation between the amplification trend of wild and mutant strains were analyzed (*R*^2^= 0.987), indicating that HMGS had accurately established the ratio in a board range of mixture proportions between drug resistant mutant isolate and the wild type *H. pylori* (**Figure 6**). Therefore, mixed infection with wild and mutant isolates could be detected and quantified by HMGS assay and predominant bacteria could be distinguished in mixed infections. We anticipated that border use of HMGS assay would improve the clinical accurate diagnosis, treatment and monitoring.

The severity of the clinical manifestations of the *H. pylori* infection was also associated with bacterial load (Kusters et al., 2006). The most regular method used for *H. pylori* quantification is RT-PCR. However, the selection of probes could easily influence the sensitivity and reproducibility of the result (He et al., 2002; Mikula et al., 2003). Moreover, RT-PCR could not amplify the target and internal control genes in the same reaction. Here, we selected the *ureC* as the quantification genes for its single copy. Meanwhile, *beta-globin* gene was introduced as internal control of human tissue that could normalize the expression of specific genes of *H. pylori*. Therefore, HMGS had a good prospect for the clinical diagnosis and treatment monitoring of *H. pylori.*

After optimizing HMGS assay in detecting *H. pylori*, as well as analyze the virulence and drug-resistance genes in isolated strains, we further explored the clinical utility of the HMGS assay for *H. pylori* diagnosis with 3 gastric biopsy specimens. Our results showed the readouts of HMGS assay performed on biopsy specimens were completely in agreement with the results of clinical diagnosis. Only human internal control beta-globin appeared in uninfected biopsy specimen (**Figure 7E**), while both *beta-globin* and the specific peaks of *H. pylori* appeared in the infected biopsy specimens (**Figures 7A,C**). Besides, the specific peaks in biopsy specimens were completely consistent with that in their corresponding isolated strains which had been confirmed by sequencing (**Figures 4A–D**). Considering the limitations of conventional detection methods for *H. pylori*, HMGS assay may efficiently skip the culture step, shorten the detection time and decrease the FNR. Based on these advantages, HMGS assay would identify *H. pylori* and detect virulence and drug resistance directly using biopsy specimens.

## Conclusion

In conclusion, we explored and optimized HMGS assay in detecting and analyzing a set of genes for *H. pylori* identification, quantification, virulence, and drug resistance in both isolated strains and biopsy specimens. Also, HMGS assay could detect mixed infections and differentiate the predominant bacteria in artificial mixed infection strains. While present study has focused on technological details and demonstration of possible clinical applications of HMGS, we believe that broad validation of its full potential will result from future studies conducted in a lager cohort of patients, possibly, at multiple study centers. We anticipate that HMGS will improve the clinical diagnosis, treatment and monitoring of *H. pylori* infection.

## Author Contributions

YZ, FZ, MK, SW contributed to this work equally. YZ, PX, YW, and HZ designed the experiments. YZ, FZ, SW, OM, and HZ wrote the manuscript.

## Conflict of Interest Statement

The authors declare that the research was conducted in the absence of any commercial or financial relationships that could be construed as a potential conflict of interest.
